# Influence of the El Niño‐Southern Oscillation on SST Fronts Along the West Coasts of North and South America

**DOI:** 10.1029/2022JC018479

**Published:** 2022-10-10

**Authors:** Caitlin M. Amos, Renato M. Castelao

**Affiliations:** ^1^ Department of Marine Sciences University of Georgia Athens GA USA; ^2^ Ocean Dynamics and Prediction Branch Naval Research Laboratory Stennis Space Center MS USA

**Keywords:** SST fronts, El Niño, California Current System, Humboldt Current System, ENSO

## Abstract

Along the west coasts of North, Central, and South America, sea surface temperature (SST) fronts are important for circulation dynamics and promoting biological activity. Prevailing equatorward winds during summer results in offshore Ekman transport and upwelling along the coast, where fronts often form between cold, upwelled water and warmer offshore waters. The interannual variability in winds, coastal upwelling, sea level anomalies, and SST in these regions have been linked to the El Niño‐Southern Oscillation (ENSO), however SST fronts have received less attention. Here, we investigate the interannual variability of SST fronts off North, Central, and South America using satellite SST data spanning 1982–2018. Anomalies of fronts within 0–300 km offshore indicate interannual variability that coincides with ENSO events in most regions. Frontal activity generally decreases during El Niño events and increases during La Niña events. The decrease in fronts off Peru and Chile during El Niño coincides with the seasonal peak in frontal activity, while off the United States the decrease occurs when frontal activity is at a seasonal minimum. We also utilized satellite measurements of wind stress and sea level anomaly to investigate how ENSO oceanic and atmospheric forcing mechanisms affect frontal activity. Decreases in frontal activity during El Niño events are largely due to oceanic forcing (i.e., coastal Kelvin waves) off Central and South America and to both oceanic forcing and atmospheric teleconnections off the United States. This study furthers our understanding of the influence of ENSO on coastal upwelling regions in the eastern Pacific Ocean.

## Key Points

- Sea surface temperature (SST) frontal activity decreases during El Niño events along most of the west coasts of North and South America
- El Niño‐related oceanic forcing impacts frontal activity off North and South America
- Atmospheric teleconnections during El Niño also affect fronts in the California Current System

## 1 Introduction

The physical, chemical, and biological processes in Eastern Boundary Current Systems (EBCS) have been widely studied over the past several decades. EBCS are associated with large‐scale anticyclonic flow, and with coastlines generally aligned in the meridional direction, these regions are favorable for coastal upwelling (Bakun & Nelson, 1991). Prevailing equatorward winds during the summer results in offshore Ekman transport and upwelling along the coast, which raises the thermocline and brings cold, nutrient‐rich waters to the surface (Huyer, 1983). Positive wind stress curl also results in upwelling, particularly ∼100–200 km from the coast, due to Ekman pumping that causes horizontal divergence at the surface, resulting in a drop in sea surface height and a corresponding rise in the thermocline (Chelton, 1982; Schwing et al., 2002). The upwelling circulation often establishes sea surface temperature (SST) fronts that separate the cold, upwelled water along the coast from warmer offshore waters (Kostianoy & Lutjeharms, 1999). A strong alongshore coastal upwelling jet also forms in geostrophic balance with the upwelled isopycnals (Huyer, 1983), making the locations of SST fronts good proxies for the location of flow intensifications in these regions (Strub & James, 2000). Fronts are also characterized by convergent flow at the surface, making them hotspots for biological activity. As free‐floating biota accumulate in the frontal zone, higher trophic levels are attracted to these regions, establishing productive food webs (Bowman, 1978; Chavez & Messié, 2009; Walsh, 1977; Woodson & Litvin, 2015).

SST fronts in EBCS have received a great deal of attention over the past two decades (e.g., Castelao et al., 2006; Mauzole et al., 2020; Meunier et al., 2012; Nieto et al., 2012; Oerder et al., 2018; Santos et al., 2012; Vazquez‐Cuervo et al., 2013; Veitch & Penven, 2017; Wang et al., 2015, 2021). In the two EBCS in the Pacific Ocean, located along the west coasts of the United States and Mexico and along Peru and Chile, fronts have been shown to exhibit latitudinal and seasonal variability, largely due to the seasonality of upwelling (Castelao & Wang, 2014; Kahru et al., 2012; Strub et al., 1998; Vazquez‐Cuervo et al., 2013; Wang et al., 2015, 2021), and mesoscale dynamics (Kahru et al., 2012; Vazquez‐Cuervo et al., 2013; Yuan & Castelao, 2017) in these regions. Interactions of the flow with bottom topography have also been shown to influence the distribution of SST fronts in many regions, such as in the California Current System (CCS; Castelao et al., 2005).

Along the west coasts of North and South America, variability in wind patterns, upwelling, sea level anomaly (SLA), and SST anomalies have been linked to climatic indices, such as the El Niño‐Southern Oscillation (ENSO) and the Pacific Decadal Oscillation (e.g., Carr et al., 2002; Enfield & Allen, 1980; Espinoza‐Morriberón et al., 2017; Huyer et al., 1987, 2002; Jacox et al., 2014, 2015; Macias et al., 2012; Meyers et al., 1998; Schwing et al., 2002; Stramma et al., 2016; Strub & James, 2002b; Strub et al., 1998; Ulloa et al., 2001). The El Niño and La Niña phases of ENSO have widespread impacts on the ocean and atmosphere, influencing the physical processes off the coasts of North, Central, and South America. Studies have identified oceanic and atmospheric mechanisms that drive changes in these regions during El Niño events. Coastally trapped waves, originating from equatorial Kelvin waves, propagate poleward along the west coasts of North and South America, depressing the thermocline and altering oceanic processes (Chelton & Davis, 1982; Enfield & Allen, 1980; Meyers et al., 1998; Shaffer et al., 1997; Spillane et al., 1987; Strub & James, 2002b). In the CCS (30–50°N), atmospheric teleconnections cause a decrease in the equatorward winds due to the expansion and displacement of the Aleutian Low to the southeast, affecting upwelling in this region (Alexander et al., 2002; Schwing et al., 2002). Off the coasts of Peru and Chile, alongshore winds are affected through different atmospheric teleconnections, which have been shown to maintain or increase upwelling favorable winds during El Niño events (Blanco et al., 2002; Carr et al., 2002; Huyer et al., 1991; Kessler, 2006).

Although a clear link has been identified between ENSO events and winds, coastal upwelling, thermocline depth, and SST anomalies along the west coasts of North and South America, the role that ENSO plays in the interannual variability of SST fronts has received less attention (Kahru et al., 2018; Wang et al., 2021). Kahru et al. (2018) used satellite SST to primarily investigate the response of fronts located between ∼22 and 38°N to the marine heatwave event in the northeast Pacific Ocean during 2014–2015 (Gentemann et al., 2017) and to the 2015–2016 El Niño. Wang et al. (2021) used 15 years of satellite SST to characterize seasonal variability of fronts along the west coasts of North, Central, and South America. They also investigated the interannual variability of fronts but only along the equator (90–135°W and 5°S–5°N) and in the coastal region between 8 and 33°N. They found that frontal activity was suppressed during El Niño years in these regions. Here, we expand on these studies by using a longer time series of SST data spanning 37 years to investigate the interannual variability of SST fronts along the west coasts of North and South America. We focus on the influence of ENSO on SST fronts in the main coastal upwelling regions in the domain, the CCS and the Humboldt Current System. We also utilize satellite measurements of SLA and wind stress to investigate the oceanic and atmospheric forcing mechanisms associated with ENSO and their effects on frontal variability.

## 2 Data and Methods

### 2.1 SST Fronts

**Figure 1:**
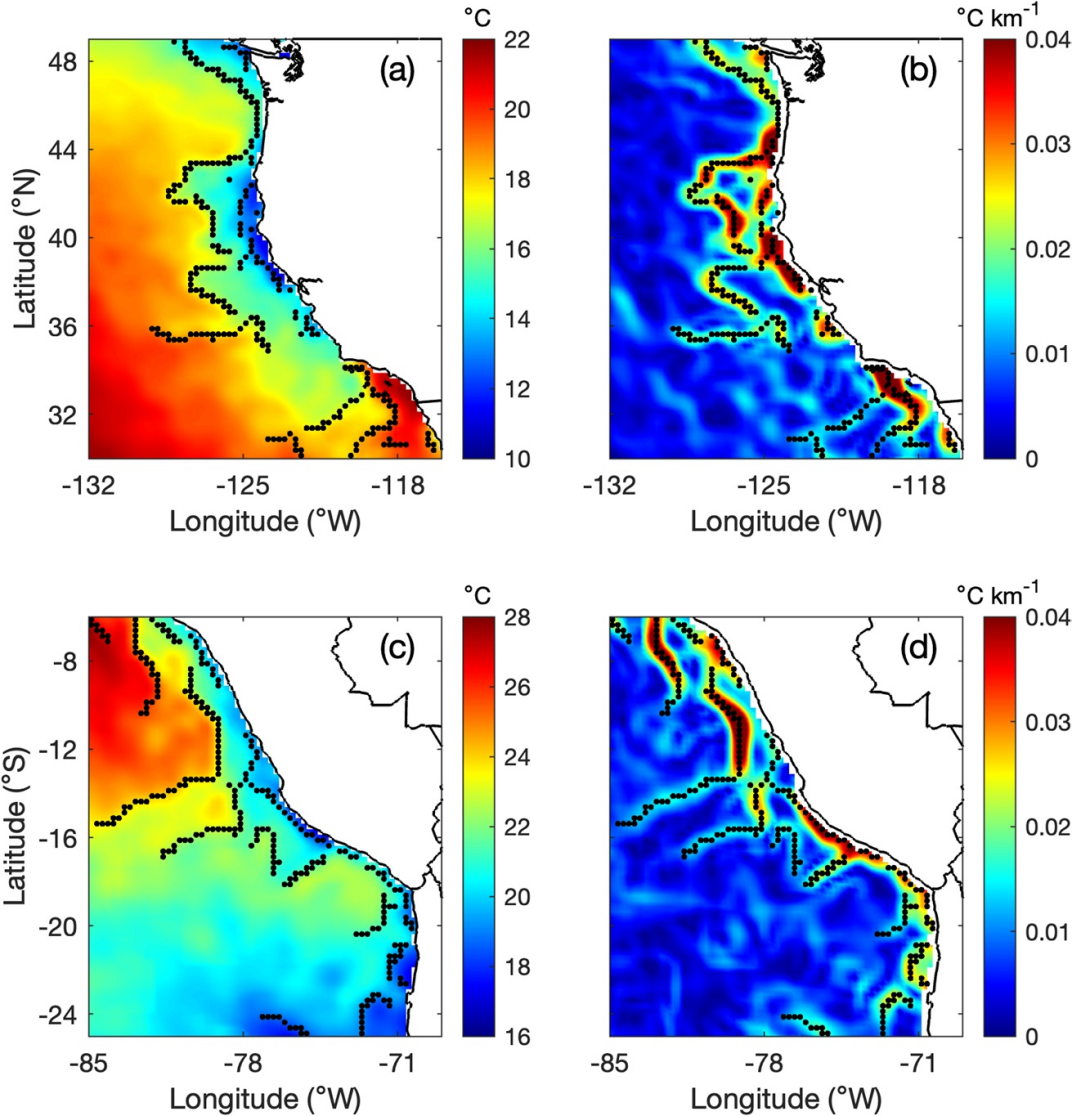
Example of using Group for High Resolution Sea Surface Temperature AVHRR‐only sea surface temperature (SST) to calculate the SST gradient magnitude and to detect fronts. (a, b) California Current System on 4 September 2016. (c, d) Humboldt Current System on 4 May 2016. (a, c) SST (°C) and (b, d) SST gradient magnitude (°C km^−1^). Black dots mark the location of fronts found by the edge‐detection algorithm.

Daily SST measurements from January 1982 to December 2018 were obtained from the Group for High Resolution Sea Surface Temperature (GHRSST). The global level 4 SST product provided by GHRSST is produced on a 0.25° grid by optimally interpolating SST observations from satellite (AVHRR only) and in situ platforms (i.e., ships and buoys). SST fronts are identified off the west coasts of North, Central, and South America using an edge‐detection algorithm (Canny, 1986) following Castelao et al. (2006) and Wang et al. (2015) (Figure 1). First, the SST gradient vector is computed for each daily SST map. The algorithm then tracks in the direction of the gradient, suppressing any pixel that is not a local maximum. The thresholding in the edge‐detection algorithm is done with hysteresis. The algorithm first looks for pixels with gradient magnitude larger than a threshold *T*_1_. These pixels are flagged as frontal pixels. The algorithm then tracks along a front crest (i.e., perpendicular to the gradient), flagging individual pixels as frontal pixels until the gradient magnitude falls below a smaller threshold *T*_2_. This helps to ensure that noisy edges are not broken up into multiple edge fragments. The thresholds employed for *T*_1_ and *T*_2_ in this study are 0.017 and 0.0085°C km^−1^, respectively. Comparisons of fronts detected with gradient magnitude maps show that the chosen threshold values allow for capturing most of the main fronts in the domain (Figure 1). Comparing the average distribution of fronts using the chosen thresholds with those from previous studies in the region (e.g., Castelao et al., 2006; Kahru et al., 2012; Wang et al., 2015) also yields consistent results. Additional details about the front detection method can be found in Castelao et al. (2006). Monthly SST frontal probabilities are calculated from the daily maps of fronts by taking the number of times each pixel (0.25 × 0.25°) qualifies as a front during 1 month and dividing by the number of times the pixel was valid (e.g., cloud free) during that month (Mavor & Bisagni, 2001; Ullman & Cornillon, 1999). GHRSST AVHRR‐only SST is cloud free due to the optimal interpolation of SST from multiple sources, however computing frontal probabilities allows for comparisons with other studies and with analyses using the Moderate‐resolution Imaging Spectroradiometer (MODIS) SST, which are described below.

One potential concern with using the GHRSST AVHRR‐only SST product is that it is characterized by a lower feature resolution compared to other SST products, such as MODIS. We repeated the analyses using daily MODIS SST measurements that are available at 4 km resolution from July 2002 to December 2018. Despite the higher spatial resolution of MODIS SST, its shorter temporal coverage is a disadvantage for studying the interannual variability of SST fronts in relation to ENSO. Analyses of fronts using GHRSST AVHRR‐only SST were consistent with the spatial and temporal patterns of fronts detected using MODIS. A regression analysis was used to compare SST gradient magnitudes (which are used to detect fronts) between the two products at monthly time scales. Along the coast of North America, the magnitude of gradients computed using GHRSST were on average 27% less than the magnitude of MODIS gradients, with the intercept centered around zero, and the correlation between the two products was, on average, 0.80. Off South America, the GHRSST gradients are on average 35% less than MODIS gradients, with the intercept centered around zero, and the average correlation was 0.69. Thus, despite the reduction in the magnitude of gradients using GHRSST AVHRR‐only SST compared to MODIS, GHRSST is still suitable for capturing SST fronts.

### 2.2 Wind Stress

The Cross‐Calibrated Multi‐Platform (CCMP) version 2.0 gridded surface vector winds from July 1987 to December 2018 are used to compute wind stress. Maps of CCMP vector winds are produced daily at 00, 06, 12, and 18Z on a 0.25° grid using a combination of Version‐7 RRS radiometer wind speeds, QuikSCAT and ASCAT scatterometer wind vectors, moored buoy wind data, and ERA‐Interim model wind fields using a Variational Analysis Method (Atlas et al., 2010). We computed wind stress for each of the four maps available for each day (00, 06, 12, and 18Z), then averaged all four maps to produce one map of wind stress for each day. The alongshore component of the wind stress was computed by rotating the coordinate system to be parallel with the local coastline. Although this product is somewhat smoothed compared to satellite wind fields (e.g., QuikSCAT and ASCAT), it has the advantage of covering a substantially longer period of time, allowing for comparisons with the GHRSST AVHRR‐only SST record.

### 2.3 Sea Level Anomaly

Sea level anomaly (SLA) data are obtained from the Copernicus Climate Service. SLA is available daily from January 1993 to December 2018 on a 0.25° grid.

### 2.4 El Niño and La Niña Events

**Figure 2:**
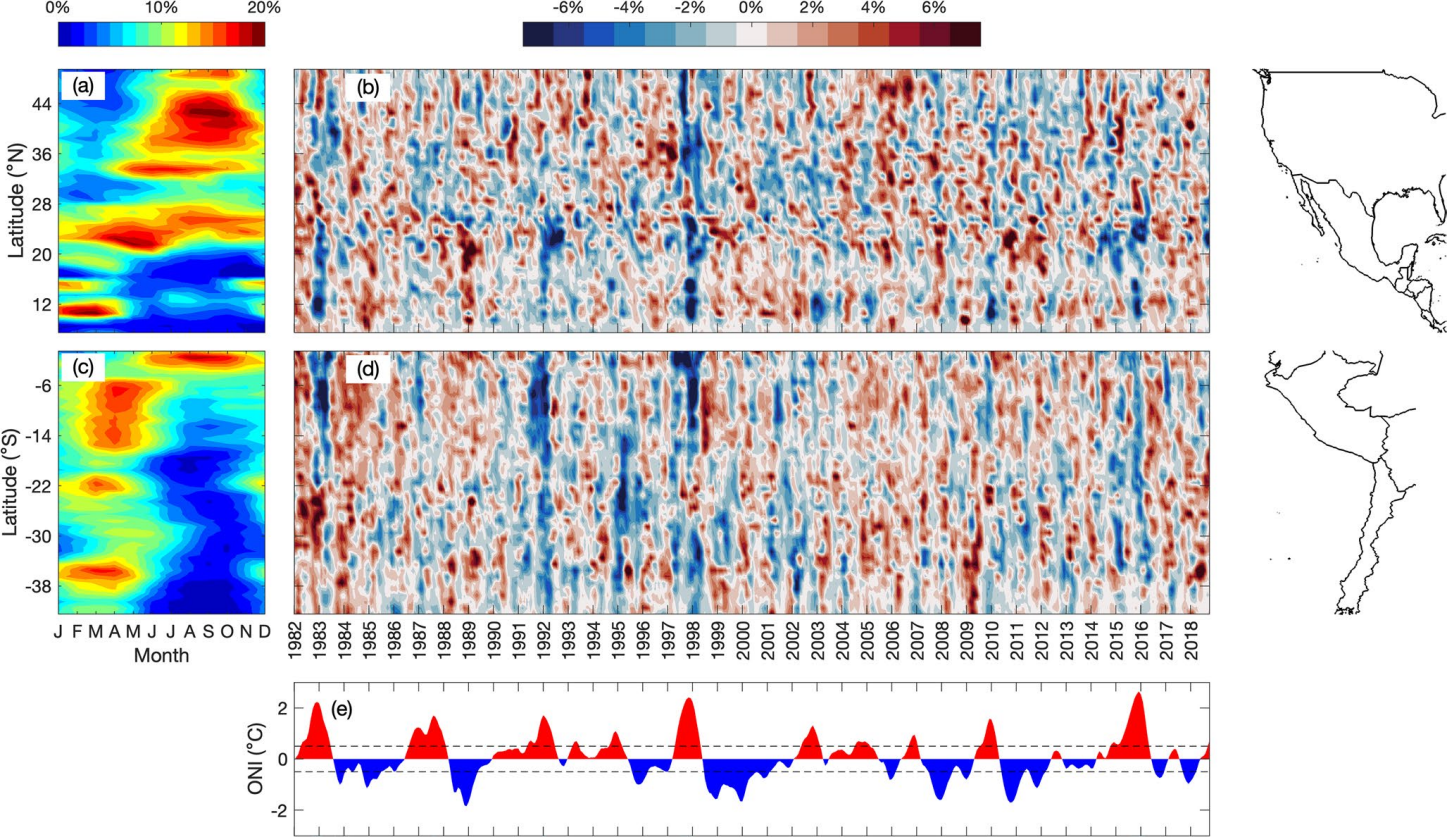
(a, c) Climatological mean (1993–2018) of sea surface temperature (SST) frontal probability (%) within 0–300 km from the coast in 1° latitude boxes, computed from fronts detected using Group for High Resolution Sea Surface Temperature AVHRR‐only SST. (b, d) The 3‐month running mean of SST frontal probability anomalies (%) computed by subtracting the climatological monthly mean (shown in panels (a and c)) and the linear trend from each individual month. (e) Oceanic Niño Index (°C) for the same time period as panels (b and d). Coastlines for North/Central America and for South America are shown on the right.

We use the Oceanic Niño Index (ONI) produced by NOAA to define El Niño and La Niña events (Figure 2e). The ONI is defined as the 3‐month running mean of SST anomalies in the Niño 3.4 region. El Niño (La Niña) events occur when the ONI reaches ≥0.5°C (≤−0.5°C) for a minimum of five consecutive months (NOAA Climate Prediction Center). Other indices are also used to define ENSO events using different parameters and/or regions of the equatorial Pacific. We repeated the analyses using the Multivariate ENSO Index, Niño 3.4, Niño 3, Niño 4, and the Southern Oscillation Index to see if the results hold true when ENSO events are defined differently.

### 2.5 Analysis Methods

Our analysis covers the west coasts of North and South America, spanning 7–50°N and 0–43°S, respectively. We divided both domains into 1° latitude boxes, extending from the coast to 300 km offshore. We chose 1° boxes so that they were large enough to include a sufficient number of data points for robust results since the horizontal resolution of the data sets is 0.25°, and to be consistent with previous studies that investigated SST fronts using 1° boxes (Castelao & Wang, 2014; Wang et al., 2015). The offshore distance was determined based on the average offshore extent of the intensity of fronts, wind stress, and SLA in the domain. The results presented here are consistent with the results obtained when using a narrower region extending for 0–100 and 0–200 km offshore. For each data set, monthly time series were computed by averaging the data within each box. Anomalies were then computed by subtracting the climatological monthly mean (1993–2018) and the linear trend from the monthly time series within each box. There was no clear linear trend for frontal probability and alongshore wind stress and a weak positive linear trend for SLA. We note that we refer to the monthly anomalies of the original SLA data as SLA anomalies. We used 1993–2018 as the climatological mean period since this was the longest common time period among all data sets. Using other climatology periods (e.g., 1993–2013) produces quantitatively similar results. To reduce noise, a 3‐month running mean was applied to the monthly anomaly time series. Lagged correlations were calculated between the smoothed anomaly time series of SST frontal probability, alongshore wind stress, and SLA in each box and the ONI. To determine significant correlations, we quantified the effective number of degrees of freedom for each data set to account for autocorrelation within the time series. We computed the number of the degrees of freedom for each data set by dividing *n* (the number of months in the time series) by the autocorrelation time scale (5 months for frontal probability and alongshore wind stress, 6 months for SLA; Lentz, 1993). Only correlations that are significant at the 95% level are presented.

Empirical orthogonal function (EOF) decompositions are utilized to isolate the dominant modes of variability (with the seasonal cycle and linear trend removed) for SST frontal probability, alongshore wind stress, and SLA. EOFs are computed using the 3‐month running mean anomaly time series in the 1° latitude boxes. To determine the statistically significant EOF modes for each data set, we used the N‐rule approach outlined by Overland and Preisendorfer (1982) to estimate those eigenvalues for which the geophysical signal exceeds the level of noise within the data. In both the Northern and Southern Hemisphere domains, the first seven modes of SST frontal probability and the first two modes of SLA were significant. For alongshore wind stress, the first three modes were significant in the Northern Hemisphere and the first five modes were significant in the Southern Hemisphere. We computed the correlation between the amplitude time series of each mode and the ONI to identify modes that were related to ENSO. The fraction of the local variance explained by each mode was computed following Davis (1976) and Chelton and Davis (1982).

To capture the progression of SST frontal probability, alongshore wind stress, and SLA during ENSO events, monthly anomalies for the 24 months covering each event (e.g., January 1997 –December 1998 to capture the 1997–1998 El Niño) were extracted from the datasets. The extracted anomalies were averaged to compute composite anomalies for all El Niño and La Niña events (Jacox et al., 2015). The composite anomalies were computed for weak events (defined as ONI between 0.5°C and 0.99°C for El Niño and between −0.99°C and −0.5°C for La Niña for at least three consecutive months) and moderate to strong events (defined as ONI ≥ 1°C for El Niño and ONI ≤ −1°C for La Niña for at least three consecutive months) to compare the response of frontal probability, alongshore wind stress, and SLA to ENSO events of different strength. A *t*‐test was used to determine the statistically significant composite anomalies at the 90% and 95% levels.

## 3 Results

### 3.1 Distribution and Interannual Variability of SST Fronts

Thirty‐seven years of SST data are used to detect SST fronts off the west coasts of North, Central, and South America. Consistent with Wang et al. (2015, 2021), SST fronts along the west coast of North America within 0–300 km offshore are most frequently observed around 11 and 15°N, along Baja California (21–30°N), and in the CCS (33–50°N) (Figure 2a). Frontal activity peaks during May–September around 33–34°N and during July–October between 37 and 45°N (Figure 2a). The highest frontal probabilities in the CCS correspond to the locations of the strongest SST gradients, which occur near irregularities in the coastline geometry (Castelao & Wang, 2014; Castelao et al., 2006; Wang et al., 2015, 2021). Along Baja California, frontal probabilities are highest during April–June and persist through December. To the south of Baja California, fronts occur in the Gulfs of Tehuantepec (15°N) and Papagayo (11°N) in boreal winter (December–March), consistent with Wang et al., 2021 (Figure 2a; referred to hereafter as “gap wind regions”). These regions are strongly influenced by intense wind jets that blow through gaps in the Sierra Madre mountain range and into the Pacific Ocean (Chelton et al., 2000; Steenburgh et al., 1998). The strong wind jets induce vertical mixing that cools the surface waters during boreal winter (Barton et al., 1993; Liang et al., 2009; Sun & Yu, 2006). SST fronts are then generated between the cold water near the coast and the surrounding warm water (Barton et al., 1993; Legeckis, 1988; Martinez‐Diaz‐De‐Leon et al., 1999; Trasviña et al., 1995).

**Figure 3:**
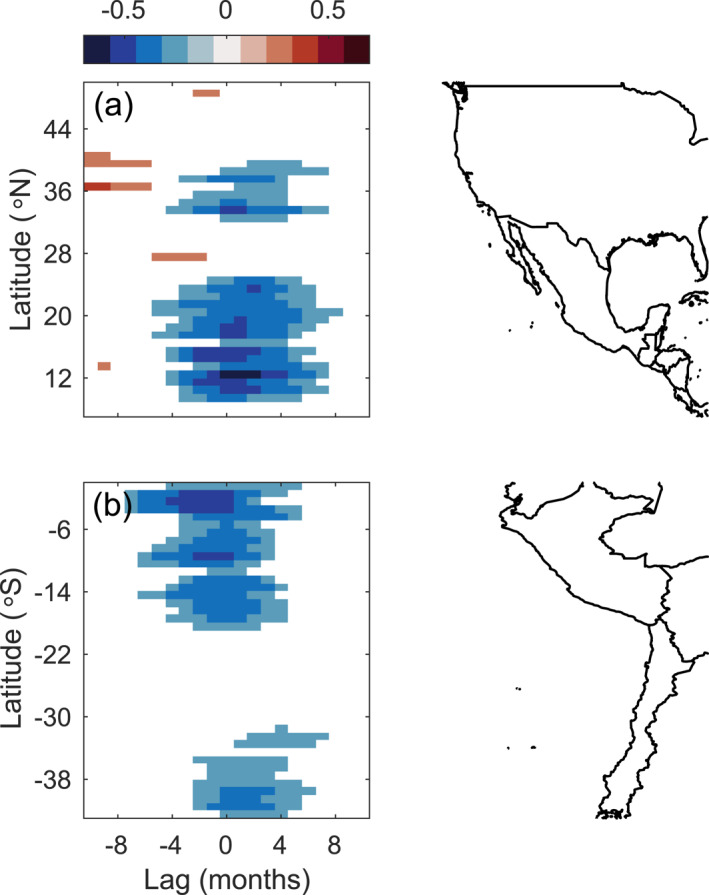
Lagged correlation between sea surface temperature (SST) frontal probability anomalies in Figures 2b and 2d and the Oceanic Niño Index (ONI) (Figure 2e) at each latitude. The *x*‐axis represents the months by which frontal probability lags the ONI (positive lag is defined as the changes in frontal probability occurring after the peak in the ONI). Only significant correlations are shown. Coastlines for North/Central America and for South America are shown on the right.

The variability in SST frontal activity that is potentially influenced by ENSO events is investigated using the monthly anomalies of frontal probabilities computed in 1° latitude boxes. Although the monthly anomalies still include variability on seasonal time scales, removing the seasonal cycle allows for interannual variability to be detected more easily. Along the west coast of North America, frontal activity generally decreases in boreal winter between 10–25°N and 33–40°N during El Niño events (Figures 2b and 2e). The anomalies of frontal probability in these regions are significantly negatively correlated with the ONI, with correlations occurring at a slightly larger lag at higher latitudes compared to the low latitude region (Figure 3a). The negative correlations imply a reduction in frontal activity during El Niño events (negative frontal probability anomalies and positive ONI during El Niño). To the north of 41°N, frontal probability decreases in boreal autumn, then increases slightly during boreal winter during El Niño events (Figure 2b), but the anomalies are not significantly correlated with the ONI (Figure 3a). The clearest signal in the anomalies and the strongest correlations with the ONI occur in the gap wind regions (11–15°N) where there is a reduction in frontal activity during boreal winter associated with El Niño events (Figures 2b and 3a), consistent with Wang et al. (2021). Anomalies of SST frontal probability were also correlated with other ENSO indices (Multivariate ENSO Index, Niño 3.4, Niño 3, Niño 4, and Southern Oscillation Index) and the results were consistent with those shown here. The magnitude of the reduction in frontal probability throughout the domain appears to be related to the strength of El Niño events. The largest reductions in frontal activity occurred during 1982–1983 and 1997–1998 (Figure 2b), coinciding with two of the strongest El Niño events during the time period covered in this study (Figure 2e). Another strong El Niño occurred in 2015–2016 and frontal activity was reduced to the south of 25°N, however in the CCS (33–50°N) there was not a clear reduction in frontal activity during boreal winter (Figure 2b).

**Figure 4:**
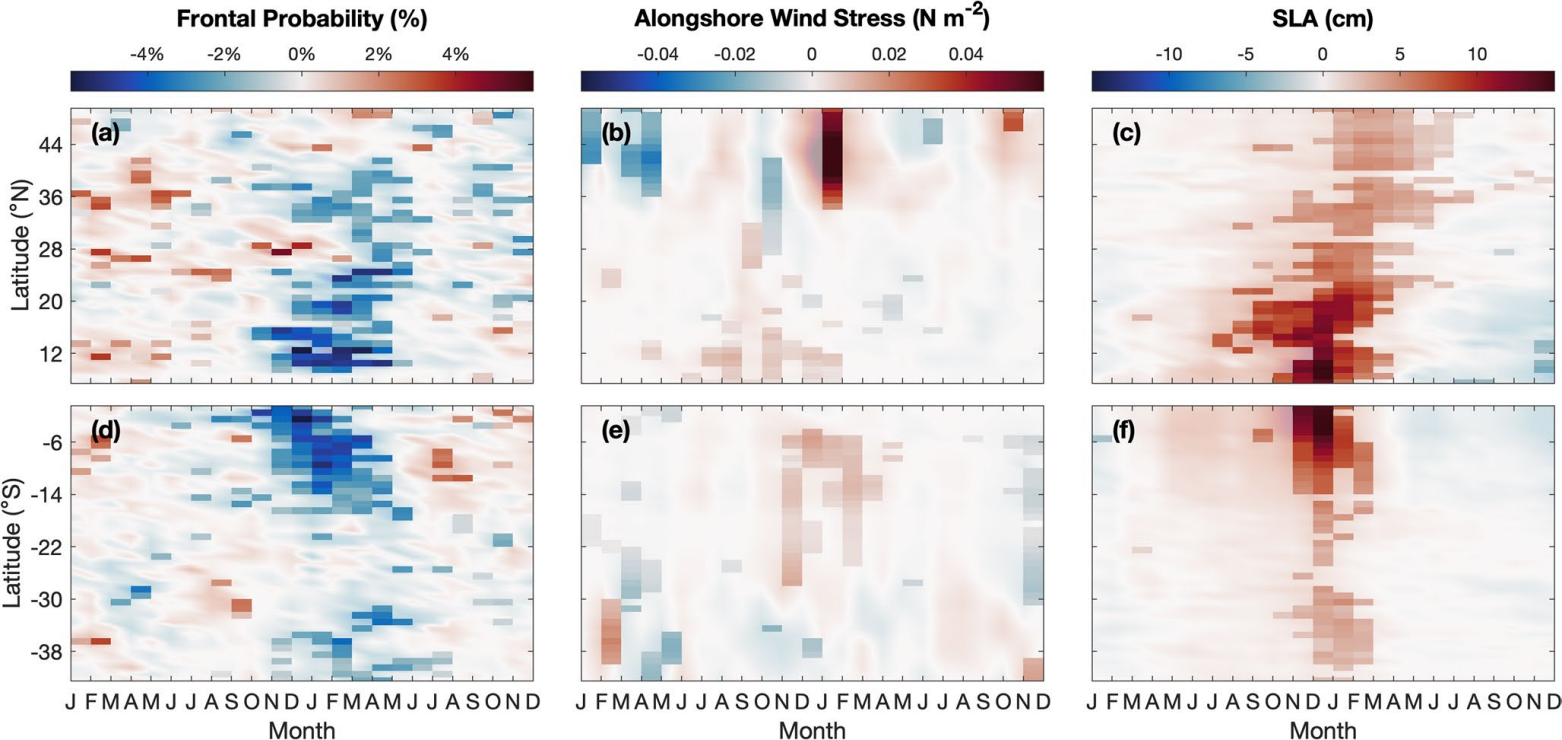
Two‐year composite anomalies for moderate to strong El Niño events (defined as Oceanic Niño Index ≥ 1°C for at least three consecutive months) in each 1° latitude box within 0–300 km offshore for (a and d) sea surface temperature frontal probability (%), (b and e) alongshore wind stress (N m^−2^), and (c and f) sea level anomaly (SLA) (cm) along the west coasts of (a, b, and d) North/Central and (d–f) South America. Light shading (transparency of 60%) indicates values that are not significant (*p*‐value > 0.1). The composites for frontal probability, alongshore wind stress, and SLA included 8, 6, and 5 El Niño events, respectively. The same data are shown in Figure S2 in Supporting Information S1, but with significance levels determined using a *p*‐value of 0.05.

**Figure 5:**
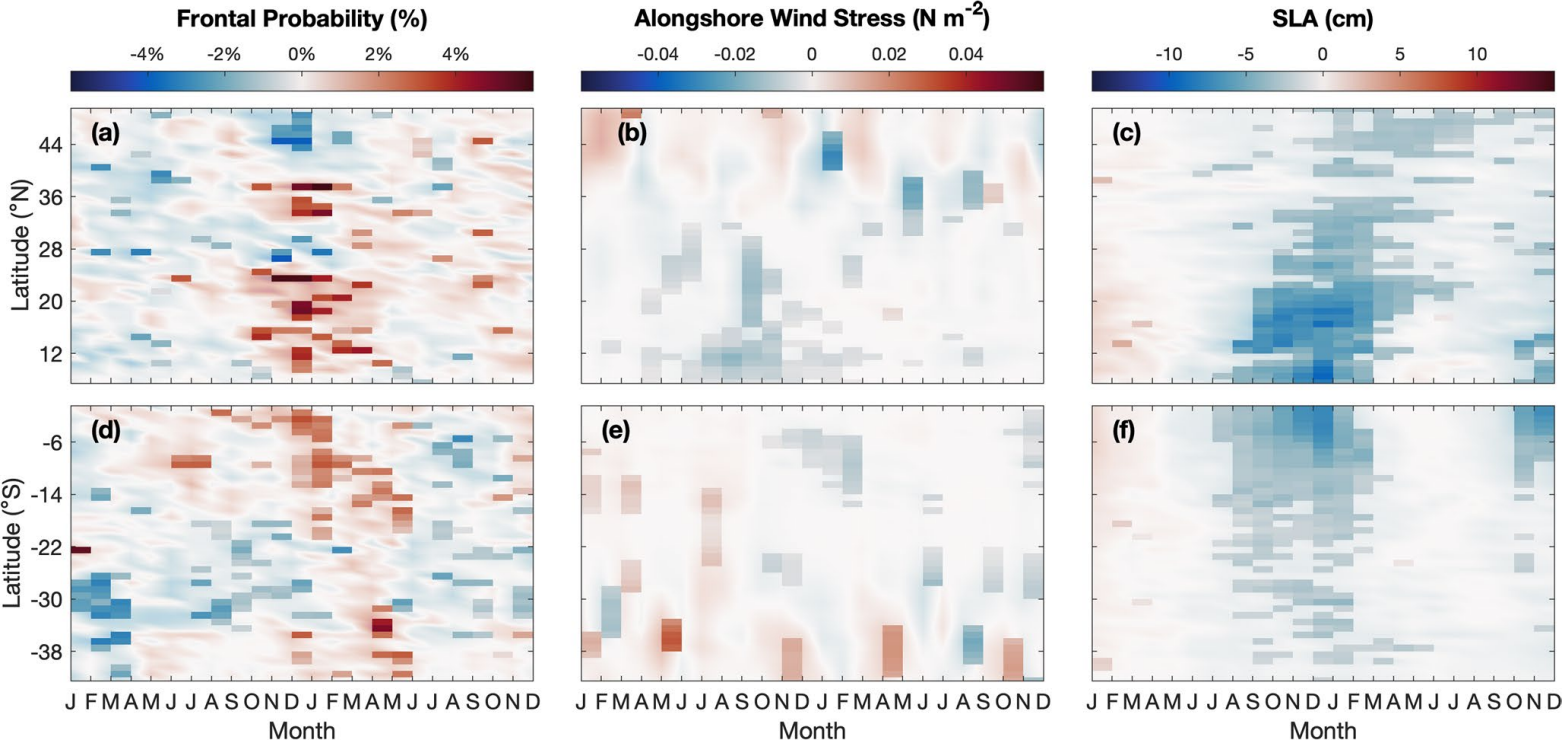
Two‐year composite anomalies for moderate to strong La Niña events (defined as Oceanic Niño Index ≤ −1°C for at least three consecutive months) in each 1° latitude box within 0–300 km offshore for (a and d) sea surface temperature frontal probability (%), (b and e) alongshore wind stress (N m^−2^), and (c and f) sea level anomaly (SLA) (cm) along the west coasts of (a, b, and d) North/Central and (d–f) South America. Light shading (transparency of 60%) indicates values that are not significant (*p*‐value > 0.1). The composites for frontal probability, alongshore wind stress, and SLA included 7, 7, and 6 La Niña events, respectively. The same data are shown in Figure S3 in Supporting Information S1, but with significance levels determined using a *p*‐value of 0.05.

These patterns are also shown in the composite anomalies of frontal probability for moderate to strong El Niño events (defined as ONI ≥ 1°C for at least three consecutive months; Figure 4a). The timing of the reduction in frontal probability in the gap wind regions (11–15°N) during boreal winter followed by reductions in the southern and central CCS (30–42°N) slightly later in the year suggests a poleward propagating signal with a speed of 75 km day^−1^ (Figure 4a; computed based on the slope of a line fitted through the largest negative anomalies during boreal winter and spring between 8 and 40°N). For weak El Niño events (defined as ONI = 0.5°C–0.99°C for at least three consecutive months), there is also a reduction in frontal probability, but the results are noisier, and the signal is less defined along the coastline compared to moderate to strong events (results not shown). During La Niña events, frontal activity increases in the gap wind regions, between 17 and 25°N, and in the southern CCS (33–39°N; Figures 2b and 5a). To the north of 43°N, frontal probability significantly decreases during November–December (Figure 5a). The magnitude of the anomalies are weaker and fewer locations and months are statistically significant for La Niña events compared to El Niño events (Figures 4 and 5).

Along the west coast of South America, SST fronts occur along the entire coastline within 0–300 km offshore (Figure 2c). Off the coast of Ecuador (0–4°S), increased frontal activity occurs during June–November, peaking around August–September (Figure 2c). Along the coast of Peru between 5 and 16°S, fronts occur during February–May (Figure 2c). To the south of 16°S, the highest frontal probabilities occur off the coast of Chile around 21–24°S and 34–39°S during February–May (Figure 2c). These spatial and seasonal patterns of fronts off the west coast of South America are consistent with Wang et al. (2015, 2021).

The anomalies of frontal probability off South America are more apparent compared to those in the Northern Hemisphere (Figure 2d). The strongest reduction in frontal activity during El Niño events occurs between the equator and ∼18°S during austral summer (Figure 2d). The frontal probability anomalies are significantly negatively correlated with the ONI in this region, with the strongest correlations occurring around 1–4°S at lags of −3 to 0 months, then the magnitude of the correlation decreases poleward (Figure 3b). To the south of 18°S, frontal activity is also often reduced during El Niño events along most of the coastline (Figure 2d), but the anomalies are only significantly correlated with the ONI between 32 and 43°S (Figure 3b). Correlating the anomalies of SST frontal probability off South America with other ENSO indices (Multivariate ENSO Index, Niño 3.4, Niño 3, Niño 4, and Southern Oscillation Index) yields results quantitatively similar to those shown here. Consistent with the Northern Hemisphere, the magnitude of the frontal probability anomalies appears to be related to the strength of the El Niño event. An exception, however, is the 1991–1992 El Niño event. This event was weaker than the El Niño events in 1982–1983 and 1997–1998 (Figure 2e), however the decrease in frontal probability in austral summer during 1991–1992 is comparable to the reductions in 1982–1983 and 1997–1998 (Figure 2d). During the 2015–2016 El Niño, frontal activity decreased less than during the events in 1982–1983 and 1997–1998, despite all three events being similar in strength based on the ONI (Figures 2d and 2e). The composite anomalies of frontal probabilities for moderate to strong El Niño events also show these general patterns (Figure 4d). Significant reductions in frontal activity occur during austral summer near the equator and slightly later in the year moving poleward (Figure 4d). During La Niña events, frontal probability generally increased during austral summer and autumn, however the signal was noisier and less significant compared to the anomalies observed during El Niño events (Figures 2d and 5d).

**Figure 6:**
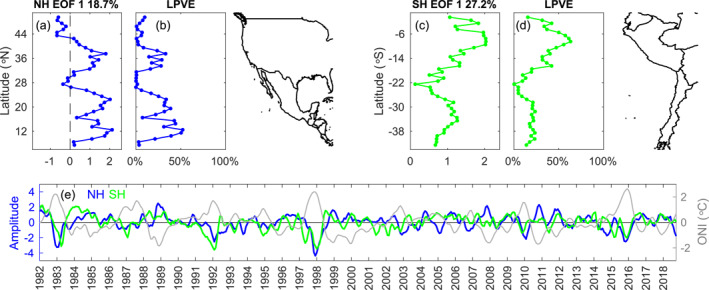
(a, c) Spatial functions, (b and d) local percentage of variance explained, and (e) amplitude time series for empirical orthogonal function 1 of sea surface temperature frontal probability anomalies (seasonal cycle and linear trend removed) along the west coasts of the Americas in the (a and b) northern and (c and d) southern hemispheres, respectively. The gray line in panel (e) is the timeseries of the Oceanic Niño Index (°C). Coastlines for North/Central America and for South America are shown on the right.

EOF decompositions of monthly SST frontal probability anomalies (seasonal cycle and linear trend removed) along the west coasts of both North and South America indicate that the dominant mode is related to ENSO events (Figure 6). We note that although a propagating signal cannot be entirely represented by one EOF mode, frontal probability anomalies associated with ENSO last for several months (especially given that a 3‐month running mean was used), allowing for the signal to be captured by the analysis. Notable decreases in the amplitude time series of EOF 1 in both hemispheres (Figure 6e) occur during El Niño events (e.g., boreal winter in 1982–1983 and 1997–1998). In the Northern Hemisphere, the amplitude time series for EOF 1 is significantly correlated with the ONI at lags of −4 to +8 months, with the peak correlation (*r* = −0.69 to −0.71) occurring at 0–2 months, which is consistent with the results in Figure 2. The spatial pattern and amplitude time series of EOF 1 indicate a reduction in frontal probability during El Niño events between 10 and 25°N and between 32 and 40°N (Figures 6a and 6e). EOF 1 accounts for a relatively small percentage of the total variance (18.7%), however it explains a larger fraction of the local variance in many regions (e.g., ∼43% at 10–16°N, ∼32% at 17–24°N, and ∼22% at 32–40°N; Figure 6b). To the north of 43°N, EOF 1 reverses sign, indicating positive anomalies of frontal probability in boreal winter during El Niño events, as shown in Figure 2b. However, mode 1 only explains ∼5% of the local variance in this region (Figure 6b).

Along the west coast of South America, EOF 1 indicates a decrease in frontal probability in austral summer during El Niño events along the entire coastline (Figures 6c and 6e). The amplitude time series for EOF 1 is significantly correlated with the ONI at lags of −5 to +5 months, with the peak correlation (*r* = −0.51 to −0.53) occurring at −1 to +1 months. EOF 1 explains 27.2% of the total variance in frontal probability, with the largest response occurring between 2 and 17°S, where the local variance explained by EOF 1 is ∼45% (Figure 6d). The amplitude time series of EOF 1 for frontal probability along the coast of South America captures a larger response in the reduction of frontal probability to weak El Niño events, for example, 1987–1988 and 1991–1992, compared to North America where the time series has a smaller amplitude during those events (Figure 6e).

### 3.2 Alongshore Wind Stress and SLA Variability

Oceanic (e.g., poleward propagating Kelvin Wave) and atmospheric (e.g., shifts in the Aleutian Low) forcing mechanisms associated with ENSO have been shown to affect winds, upwelling, thermocline depth, and SST along the west coasts of North, Central, and South America (e.g., Carr et al., 2002; Enfield & Allen, 1980; Espinoza‐Morriberón et al., 2017; Huyer et al., 1987, 2002; Jacox et al., 2014, 2015; Macias et al., 2012; Schwing et al., 2002; Stramma et al., 2016; Strub & James, 2002b; Strub et al., 1998; Ulloa et al., 2001). Therefore, it is useful to investigate the variability in alongshore wind stress and SLA to understand how these forcing mechanisms may affect SST frontal activity.

**Figure 7:**
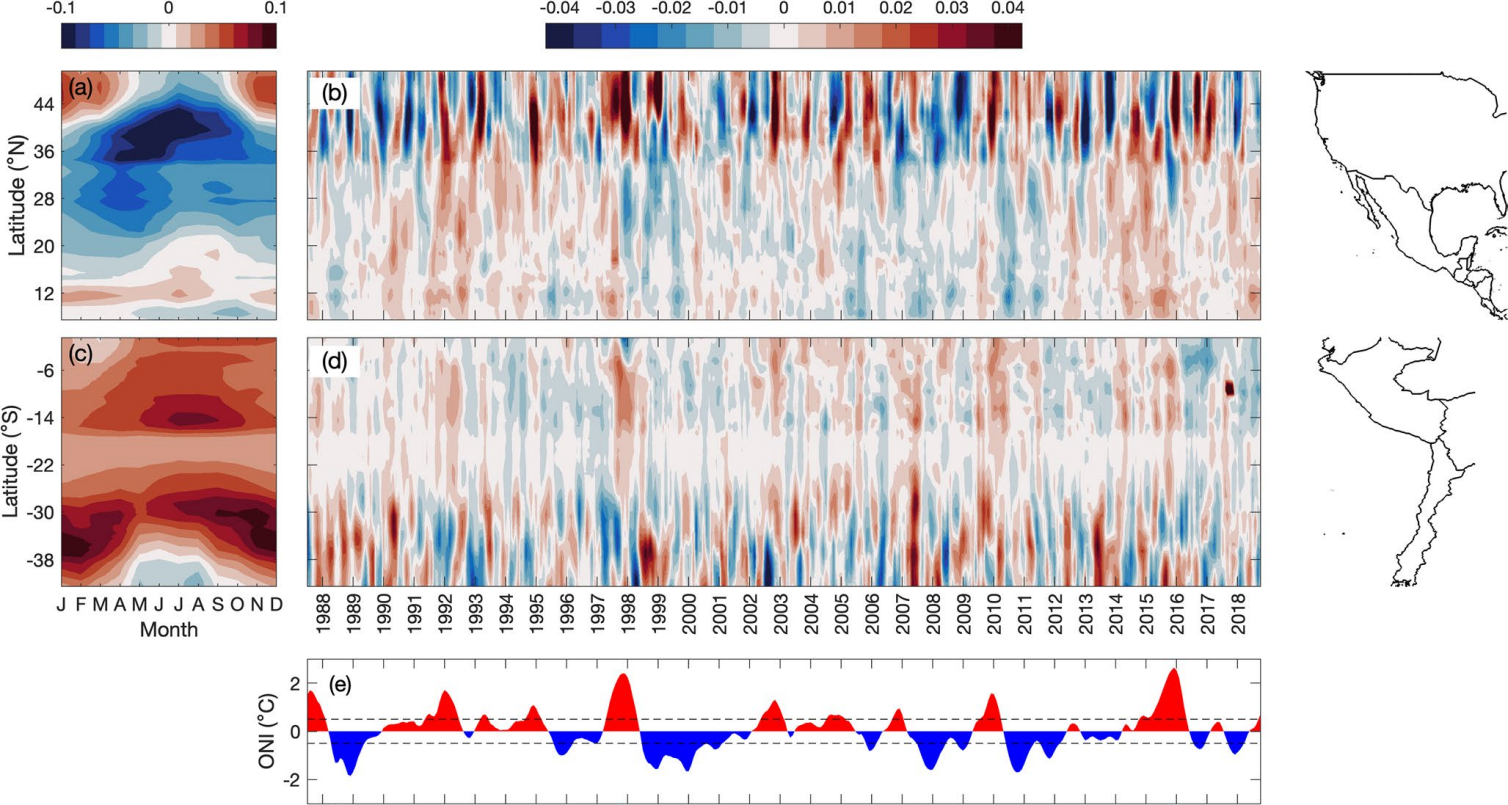
(a, c) Climatological mean (1993–2018) of alongshore winds stress (N m^−2^) averaged within 0–300 km from the coast in 1° latitude boxes, computed using Cross‐Calibrated Multi‐Platform version 2.0 surface vector winds. (b, d) The 3‐month running mean of alongshore wind stress anomalies (N m^−2^) computed by subtracting the monthly climatological mean (shown in panels (a and c)) and the linear trend from each individual month. (e) Oceanic Niño Index (°C) for the same time period as panels (b and d). Coastlines for North/Central America and for South America are shown on the right.

Along the west coast of North America, negative alongshore wind stress (upwelling favorable in the Northern Hemisphere) within 0–300 km offshore is dominant to the north of 20°N (Figure 7a). Alongshore wind stress off California (34–39°N) is upwelling favorable year‐round on a monthly time scale, with peak intensity during April–June (Figure 7a). To the north of 39°N, wind stress is upwelling favorable during May–September and downwelling favorable during November–March (Figure 7a). These patterns in the alongshore wind stress in the CCS are consistent with Huyer (1983), Strub et al. (1987), Chelton et al. (2007), and García‐Reyes and Largier (2012). To the south of 20°N, cross‐shore wind stress becomes more important (not shown) due to the gap wind regions where intense wind jets blow through gaps in the Sierra Madre mountain range into the Pacific Ocean around 11 and 15°N during boreal winter (Chelton et al., 2000; Steenburgh et al., 1998).

Monthly anomalies of the alongshore wind stress within 0–300 km of the coast off North America are most pronounced to the north of 34°N. The anomalies are generally positive in the CCS (34–48°N) in boreal winter during El Niño events (e.g., 1997–1998 and 2002–2003; Figures 7b and 7e), indicating more downwelling favorable or less upwelling favorable wind stress, consistent with previous studies (Strub & James, 2002a; Schwing et al., 2002). The anomalies are weaker in magnitude to the south of 34°N (Figure 7b), which is consistent with the spatial pattern of alongshore wind stress discussed above in which alongshore wind stress is most pronounced to the north of 34°N (Figure 7a). These patterns are also shown in the 2‐year composite anomalies of alongshore wind stress for moderate to strong El Niño events (Figure 4b). Alongshore wind stress anomalies significantly increased during January–February in the CCS and there were much weaker and mostly non‐significant anomalies to the south of ∼34°N for most months (Figure 4b). During La Niña events, there are alternating bands of weakly positive and weakly negative anomalies in the CCS during boreal winter (Figures 5b and 7b). The composite anomalies for moderate to strong La Niña events indicate that the anomalies throughout most of the region and during most months are not significant (Figure 5b).

Along the west coast of South America, positive alongshore wind stress (upwelling favorable in the Southern Hemisphere) persists year‐round between 0 and 35°S (Figure 7c). Upwelling favorable winds peak during June–September to the north of 16°S along the coast of Ecuador and Peru and are continuously strong almost year‐round between 28 and 35°S, consistent with Putrasahan et al. (2013). To the south of ∼36°S, positive wind stress occurs during October–April and switches to negative (downwelling favorable in the Southern Hemisphere) during May–August (Figure 7c), consistent with Strub et al. (2019).

The monthly anomalies of alongshore wind stress are weakly positive during October–May between 5 and 20°S during El Niño events, with the anomalies extending farther south during some events (e.g., 1997–1998 and 2009–2010; Figures 7d and 7e). This indicates maintained or increased upwelling favorable wind stress during El Niño events, which is consistent with previous studies (Blanco et al., 2002; Carr et al., 2002; Huyer et al., 1991). During moderate to strong El Niño events, the magnitude of the anomalies at higher latitudes are lower compared to the CCS (Figure 4e). During La Niña events, anomalies off the coast of South America are quite small (Figure 7d). Composite anomalies for moderate to strong La Niña events indicate very weak negative anomalies during austral summer throughout most of the domain, with significant anomalies occurring between 7 and 14°S during November–March and only a few significant anomalies to the south of 18°S (Figure 5e).

**Figure 8:**
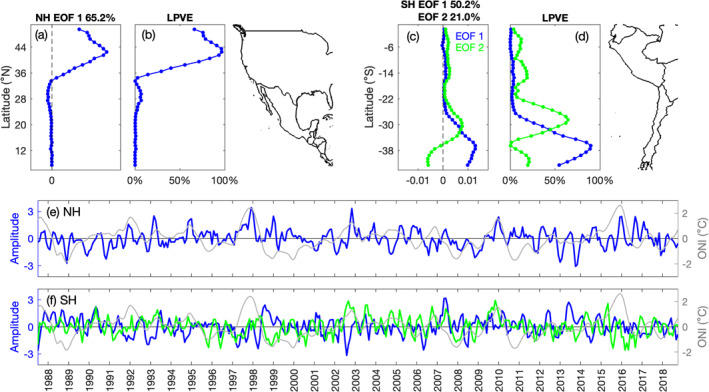
(a, c) Spatial functions, (b and d) local percentage of variance explained, and (e and f) amplitude time series for empirical orthogonal function (EOF) 1 of alongshore wind stress anomalies (seasonal cycle and linear trend removed) along the west coasts of the Americas in the (a, b, and e) northern and (c, d, and f) southern hemispheres respectively. EOF 2 for alongshore wind stress off South America is shown in green in panels (c, d, and f). The gray line in panels (e and f) is the timeseries of the Oceanic Niño Index (°C). Coastlines for North/Central America and for South America are shown on the right.

EOF 1 of monthly alongshore wind stress anomalies (with the seasonal cycle and linear trend removed) captures the dominant mode of variability along the west coast of North America, which is likely associated with ENSO, as indicated by signals in the amplitude time series that coincide with El Niño events (Figure 8e). EOF 1 accounts for 65.2% of the total variance and specifically captures variability in alongshore wind stress in the CCS (35–50°N), where the local variance explained by EOF 1 ranges from 30% to 97% (Figure 8b). The amplitude time series and spatial function for EOF 1 indicate positive alongshore wind stress anomalies (more downwelling favorable or less upwelling favorable) in the CCS that peak during December–February during El Niño events (Figures 8a and 8e), which is consistent with the results in Figure 7b. The amplitude time series is significantly correlated with the ONI at lags of −1 to +1 months (*r* = 0.22 to 0.24; positive lag is defined as changes in alongshore wind stress occurring after the peak in the ONI). To the south of 34°N, EOF 1 captures only ∼1.5% of the local variance of alongshore wind stress (Figure 8b). EOF modes 2 and 3 explain more of the local variance in this region (not shown), however the amplitude time series are not significantly correlated with the ONI.

Along the west coast of South America, EOF 1 primarily captures variability in the anomalies of alongshore wind stress between 28 and 43°S (Figure 8c). Although EOF 1 accounts for 50.2% of the total variance and explains 11%–90% of the local variance between 28 and 43°S, the amplitude time series does not show a clear pattern coinciding with El Niño and La Niña events and it is not significantly correlated with the ONI (Figure 8f). ENSO variability in the alongshore wind stress off the coast of South America is likely captured by EOF 2. The amplitude time series for EOF 2 is significantly correlated with the ONI at lags of −1 to 0 months (*r* = 0.22). The spatial pattern and amplitude time series for EOF 2 indicate small positive alongshore wind stress anomalies (more upwelling favorable in the Southern Hemisphere) between 2 and 16°S and larger positive anomalies between 23 and 34°S during El Niño events, spanning austral spring and summer (Figures 8c and 8f). To the south of 35°S, the spatial function reverses sign (Figure 8c), indicating negative alongshore wind stress anomalies (less upwelling favorable) during El Niño events. EOF 2 captures 21.0% of the total variance and ∼12% (0–17°S), 24%–64% (23–34°S), and ∼20% (38–43°S) of the local variance of alongshore wind stress anomalies (Figure 8d). The results from EOF 1 for the Northern Hemisphere and EOF 2 for the Southern Hemisphere are consistent with the Hovmöller plots of alongshore wind stress anomalies (Figure 7), showing that upwelling favorable wind stress generally decreases along the west coast of the United States and increases off the coast of Peru in boreal winter (austral summer) during El Niño events.

Consistent with previous studies (e.g., Carr et al., 2002; Chelton & Davis, 1982; Enfield & Allen, 1980; Strub & James, 2002b), monthly SLA anomalies are positive within 0–300 km offshore along the west coasts of North and South America during El Niño events (Figure S1 in Supporting Information S1). The timing and spatial extent of the SLA signal is clearly shown in the composite anomalies for moderate to strong El Niño events (Figures 4c and 4f). In the Northern Hemisphere, the positive SLA anomalies associated with El Niño events are significant along most of the coastline, with the largest increase occurring to the south of ∼22°N and peaking in magnitude around December (Figure 4c). The SLA signal persists to 50°N but gradually loses strength and slightly lags behind the signal to the south. This indicates a poleward propagating signal with a speed of ∼75 km day^−1^ that is consistent with previous studies (Chelton & Davis, 1982; Clarke & Van Gorder, 1994; Enfield & Allen, 1980). Note that this propagation speed is identical to the propagation speed identified previously based on the SST frontal probability anomalies. South of the equator, the positive SLA anomalies are also significant along most of the coastline (Figure 4f). The largest signal occurs between 0 and 10°S during November–December and quickly decreases in magnitude to the south of 10°S. The SLA signal along the west coast of South America travels much faster than the signal along the coast of North America, at a speed of ∼200 km day^−1^ (Figure 4f, Shaffer et al., 1997; Spillane et al., 1987). During La Niña events, SLA anomalies were negative along both coastlines, however the magnitude of the anomalies was reduced compared to El Niño events (Figures 5c, 5f, and Figure S1 in Supporting Information S1). Similar to El Niño events, the largest anomalies occur at lower latitudes off both North and South America (7–20°N and 0–8°S, respectively) during moderate to strong La Niña events and the signal generally persists to higher latitudes but loses strength (Figures 5c and 5f).

## 4 Discussion and Conclusions

The ENSO can have widespread impacts on the ocean and atmosphere, influencing physical and biological processes. Over the past several decades, studies have identified oceanic and atmospheric forcing mechanisms associated with ENSO that drive changes in winds, coastal upwelling, thermocline depth, and SST along the west coasts of North, Central, and South America (e.g., Carr et al., 2002; Enfield & Allen, 1980; Espinoza‐Morriberón et al., 2017; Huyer et al., 1987, 2002; Jacox et al., 2014, 2015; Macias et al., 2012; Meyers et al., 1998; Schwing et al., 2002; Stramma et al., 2016; Strub & James, 2002b; Strub et al., 1998; Ulloa et al., 2001). Here, we investigated the influence of ENSO specifically on SST fronts in these regions. After removing the seasonal cycle and linear trend, the variability in frontal activity is dominated by ENSO in both hemispheres (Figure 6). SST frontal activity generally decreases during boreal winter and early spring (austral summer and early autumn) during El Niño events and increases during La Niña events in most of the domain, with the magnitude of the anomalies generally following the strength of the events (Figures 2 and 4, 5, 6).

Recent studies investigated the variability of SST fronts along the coast of Baja California to the southern CCS (Kahru et al., 2018) and along the entire west coasts of North, Central, and South America (Wang et al., 2021). Kahru et al. (2018) used MODIS and VIIRS satellite data spanning 2000–2017 to detect SST fronts between ∼22 and 38°N. They were primarily focused on the response of fronts to the marine heatwave event in the northeast Pacific Ocean during 2014–2015 (Gentemann et al., 2017) and to the 2015–2016 El Niño. Wang et al. (2021) used MODIS SST spanning 2002–2017 to detect SST fronts along the west coasts of North and South America. They mainly focused on characterizing the seasonal evolution of fronts and its relation to wind forcing and only investigated the interannual variability associated with ENSO in the tropical Pacific Ocean (90–135°W, 5–5°N) and along Central America and Mexico (8–33°N). Both of these studies use satellite SST datasets that only captured variability in SST fronts since the early 2000s, which misses the strong El Niño events in 1982–1983 and 1997–1998. Our analysis expands on these studies by using a longer time period of SST data (1982–2018) to include more ENSO events, allowing us to capture the variability of fronts in response to numerous El Niño events of varying strength. Our results are generally consistent with Kahru et al. (2018) for the 2015–2016 El Niño, showing a slight reduction in SST frontal probability in part of the region between 22 and 38°N (see Figure 6 in Kahru et al., 2018). Wang et al. (2021) found significant negative correlations between frontal probability and ENSO in the gap wind regions (11–15°N; regions 3–4 in Wang et al., 2021) and along Mexico (16–32°N; region 2 in Wang et al., 2021) when ENSO leads frontal probability by −4 to +4 months and −5 to +1 months, respectively. The negative correlations indicate a decrease (increase) in frontal probability during El Niño (La Niña) events. Our results are consistent with Wang et al. (2021) in the gap wind regions and along the southern half of Mexico (18–25°N) where we also found significant negative correlations between frontal probability and ENSO at −4 to +4 months of lag. However, our results differ along the northern half of Mexico (26–32°N) where we did not find significant negative correlations. This difference is likely due to differences in the regions used in the analyses. We used 1° latitude boxes extending 0–300 km offshore for our analyses, so we are able to capture alongshore variability in the relationship between frontal activity and ENSO in higher resolution, whereas Wang et al. (2021) used one box covering ∼17–32°N and a farther offshore distance. Our study also expands on Wang et al. (2021) by investigating interannual variability of fronts off the United States and South America and the influence of ENSO forcing mechanisms on frontal variability.

As discussed, frontal activity generally decreases during El Niño events along most of the west coasts of North, Central, and South America. An exception to this is in the middle of both domains, between 25 and 32°N and 19–32°S, where frontal activity is not significantly correlated with the ONI. Another difference is observed during the 2015–2016 El Niño. This event became the strongest El Niño since the 1997–1998 event based on SST anomalies in the equatorial Pacific (Figure 2e), however the reduction in frontal probability was smaller than expected given the strength of the event and the response of fronts observed during the strong events in 1982–1983 and 1997–1998 (Figures 2 and 6). Monthly anomalies of SLA indicated that the maximum anomalous SLA values at lower latitudes were reduced by about 30% off Central America and southern Mexico (7–20°N), by 54% off Ecuador and Peru (0–18°S), and by 48% off Chile (18–43°S) during the 2015–2016 El Niño compared to the 1997–1998 event. Strub et al. (2019) also calculated a reduction in maximum SLA anomalies at lower latitudes along South America by approximately half in 2015 compared to 1997. In the lower latitude regions, where atmospheric forcing is weak compared to higher latitudes, the smaller response of frontal probability to the 2015–2016 El Niño may be due to weaker oceanic forcing (e.g., less depression of the thermocline) that occurred during this event. In the CCS, the thermocline was shallower than what was observed during the 1982–1983 and 1997–1998 El Niño events and there was less weakening of upwelling favorable winds (Figure 7; Jacox et al., 2016). There was also a marine heatwave in the northeast Pacific Ocean during 2014–2016 that caused unusually warm SST anomalies and other impacts in the CCS (Gentemann et al., 2017). Additionally, the 2015–2016 El Niño has been classified as a different El Niño flavor than the 1982–1983 and 1997–1998 events (Paek et al., 2017; Santoso et al., 2017). Recent studies have identified two dominant flavors of ENSO: central Pacific and eastern Pacific types (Kao & Yu, 2009; Takahashi et al., 2011). The eastern Pacific type of ENSO is characterized by SST anomalies centered in the eastern equatorial Pacific Ocean and extending to the west coast of South America and with thermocline and surface wind variations occurring basin wide. The central Pacific type of ENSO has most of its surface wind, SST, and subsurface anomalies confined in the central Pacific Ocean and appears less related to thermocline variations and may be influenced more by atmospheric forcing (Kao & Yu, 2009; Takahashi et al., 2011). Paek et al. (2017) showed that the 2015–2016 El Niño developed from a combination of eastern Pacific and central Pacific El Niño dynamics, while the 1997–1998 El Niño developed following only eastern Pacific dynamics. The central Pacific component of the 2015–2016 event caused SST anomalies to occur more westward in the central Pacific and farther from the coast of South America (Paek et al., 2017). As a result, these changes in the oceanic forcing, in addition to potential changes in atmospheric forcing, may have caused smaller SST anomalies along the coasts of North and South America, resulting in the smaller decrease in frontal probability observed in 2015–2016 compared to 1982–1983 and 1997–1998. The variability in the response of frontal probability to various ENSO events and the weaker signal in the reduction in frontal probability off the west coasts of North and South America during the 2015–2016 El Niño may be due to a combination of changes in both oceanic and atmospheric conditions that differed from the other strong El Niño events, in addition to the different flavors of El Niño events discussed above.

The ENSO‐related forcing mechanisms likely affect frontal activity along the west coasts of North and South America through atmospheric and oceanic pathways. Off the west coast of the United States, alongshore wind stress becomes more downwelling favorable during boreal winter to the north of 35°N during El Niño events (Figure 4). These anomalies are consistent with atmospheric teleconnections during El Niño events that strengthen and extend the Aleutian low to the southeast, causing equatorward winds (upwelling favorable) to weaken and shift to poleward winds in the CCS (Alexander et al., 2002; Schwing et al., 2002). More downwelling favorable (or less upwelling favorable) wind stress can reduce upwelling, decreasing the surface temperature gradient and inhibiting the formation of SST fronts. SST frontal probability decreased in the CCS in boreal winter during El Niño events, coinciding with the increase in downwelling favorable wind stress. Alongshore winds along the coast of South America to the north of 33°S remained upwelling favorable in austral summer during El Niño events. This is consistent with previous studies showing that ENSO‐related atmospheric forcing often causes alongshore winds to remain upwelling favorable during El Niño events along the coasts of Peru and Chile (Blanco et al., 2002; Carr et al., 2002; Huyer et al., 1991; Kessler, 2006). Despite persistent upwelling favorable winds in austral summer during El Niño events, frontal probability decreased during this time. This suggests that other ENSO forcing mechanisms are responsible for the reduction in frontal activity off South America (see discussion below).

Frontal activity may also be influenced by ENSO‐related oceanic forcing. During El Niño events, coastal Kelvin waves that propagate poleward along the west coasts of Central/North and South America are generated (Chelton & Davis, 1982; Enfield & Allen, 1980; Meyers et al., 1998; Shaffer et al., 1997; Spillane et al., 1987; Strub & James, 2002b). As these waves propagate poleward, the thermocline deepens along the coast (Blanco et al., 2002; Chavez et al., 2002; Frischknecht et al., 2015; Huyer et al., 1987), and SLA becomes positive (Chelton & Davis, 1982; Enfield & Allen, 1980; Meyers et al., 1998; Shaffer et al., 1997; Spillane et al., 1987; Strub & James, 2002b). This is accompanied by a decrease in frontal probability, suggesting a possible connection to ENSO‐related oceanic forcing along both coastlines.

Given the spatial patterns of wind stress and SLA anomalies that occur along the west coasts of North and South America during El Niño events, the ENSO‐related atmospheric and oceanic forcing mechanisms likely contribute differently to the observed decrease in frontal activity in different regions along the coasts. The largest SLA signals during El Niño events occurs at lower latitudes in both hemispheres, suggesting oceanic forcing (i.e., coastal Kelvin waves) likely has a larger influence on frontal activity in these regions compared to higher latitudes. In the gap wind regions (11–15°N), where winds blowing offshore dominate in boreal winter, Romero‐Centeno et al. (2003) and Karnauskas et al. (2008) showed that these winds increase during El Niño events. Presumably, this increase in the offshore‐directed wind stress would increase the vertical mixing that causes the localized cooling of the surface waters (Barton et al., 1993; Liang et al., 2009; Sun & Yu, 2006) and the formation of fronts (Barton et al., 1993; Legeckis, 1988; Martinez‐Diaz‐De‐Leon et al., 1999; Trasviña et al., 1995), resulting in an increase in frontal activity. However, frontal activity actually decreased in these regions during El Niño events, at a time when SLA anomalies were positive. Alexander et al. (2012) showed that ENSO‐driven warming in the gap wind regions was associated with deepening of the thermocline due to coastal Kelvin waves, rather than atmospheric anomalies. Farther south along the west coast of South America between 5 and 33°S, alongshore wind stress anomalies are positive (i.e., upwelling favorable) during El Niño events, yet frontal probability decreases. The decrease in frontal probability coincides with positive SLA anomalies along the coast of South America. Therefore, the influence of ENSO‐related oceanic forcing likely outweighs the effects of atmospheric forcing on frontal activity in the gap wind regions and at low‐ to mid‐latitudes regions off South America. In the CCS (35–50°N), where alongshore wind stress anomalies are large but the coastal Kelvin wave signal persists, it is difficult to identify which forcing mechanism (atmospheric or oceanic) has a larger influence on the reduction in front activity based on observations alone. Future studies using idealized model simulations where forcing is systematically varied could help disentangle the contribution of different forcing mechanisms associated with ENSO on frontal variability in the region.

It is important to note the timing of the reduction in frontal activity during El Niño events in relation to the timing of peak frontal activity. SST frontal probability peaks during boreal summer along the west coast of the United States (33–50°N) and during boreal winter (austral summer) off southern Mexico (7–20°N), Central America, and South America to the south of 5°S. Thus, along the west coasts of southern Mexico, Central America, and Peru and Chile (5–43°S) the decrease in frontal probability in austral summer (boreal winter) during El Niño events occurs when frontal activity is highest in these regions. This also aligns with the timing of peak El Niño conditions in the equatorial Pacific. Along the west coast of the United States, on the other hand, frontal probability also decreases in boreal winter during El Niño events, but frontal activity is lowest during this time of year. This out of phase response at higher latitudes in the Northern Hemisphere between the reduction in frontal probability during El Niño events and the timing of peak frontal activity, compared to the in‐phase response along South America, may contribute to EOF 1 of frontal probability anomalies explaining more of the total variance in the Southern Hemisphere than in the Northern Hemisphere.

The west coasts of North and South America feature very productive ecosystems during local summer when upwelling intensifies and frontal activity peaks (Carr, 2001; Carr & Kearns, 2003; Chavez & Messié, 2009). Fronts become hotspots for biological activity due to flow convergence that causes free‐floating biota to accumulate, which attracts higher trophic levels and establishes productive food webs (Bowman, 1978; Chavez & Messié, 2009; Walsh, 1977; Woodson & Litvin, 2015). ENSO may impact biological activity due to the reduction in fronts observed during El Niño events. This impact may be felt more along the coast of Central and South America since the largest reduction in frontal activity during El Niño occurs in austral summer (boreal winter) when fronts are most frequently observed in those regions. Our results also suggest that ENSO may affect the strength of ocean‐atmosphere interactions along the west coasts of North and South America. Air‐sea heat fluxes affect stability and mixing in the lower atmosphere, causing wind stress to increase over warm water and decrease over cold water (Chelton et al., 2001, 2007). In the vicinity of SST fronts, the change in wind stress as winds blow over the warm and cold sides of the fronts generates wind stress curl and divergence that are linearly related to the crosswind and downwind components of the SST gradient, respectively (Chelton et al., 2001, 2007). The coupling between SST gradients and wind stress curl and divergence peaks during boreal summer off the west coast of the United States (Chelton et al., 2007; Wang & Castelao, 2016) and during boreal winter (austral summer) off Central America, Peru and Chile (Wang & Castelao, 2016). The changes in frontal activity during ENSO events may affect this coupling differently in these regions. Specifically, ENSO will possibly have a larger influence modulating air‐sea coupling along the coast of Central and South America since the reduction in frontal activity during El Niño events coincides with peak frontal activity and stronger air‐sea coupling. Along the west coast of the United States, on the other hand, ENSO may have less impact on air‐sea coupling associated with fronts due to the out of phase response between the reduction in frontal activity during El Niño events and the timing of peak frontal activity and stronger air‐sea coupling.

The anomalies for frontal probability, alongshore wind stress and SLA during La Niña events are generally of opposite sign compared to El Niño events along both coastlines, but the magnitude of the anomalies is less (Figures 4 and 5). These results are consistent with other studies showing opposite but reduced anomalies in SST, SLA, and upwelling conditions off North and South America during La Niña events compared to El Niño events (e.g., Carr et al., 2002; Jacox et al., 2015; Schwing et al., 2002; Smith et al., 2001). These slight asymmetries in the anomalies may be associated with spatial and temporal asymmetries of El Niño and La Niña which are caused by various nonlinear processes (e.g., nonlinear ocean advection, nonlinear atmosphere‐ocean coupling, tropical instability waves, etc.; McPhaden et al., 2020). Strong El Niño events are generally centered along the equator over the eastern Pacific, whereas strong La Niña events are mostly centered over the central Pacific with a wider latitudinal extension (McPhaden et al., 2020). The amplitude of El Niño events is usually greater than that of La Niña and decays rapidly after the peak of the event, while La Niña events often persist for longer periods (Figure 2e). These spatial and temporal asymmetries likely affect the atmospheric and oceanic forcings associated with ENSO events, potentially causing some of the asymmetries seen in our results. The idealized modeling simulation mentioned above could also be used to investigate potential asymmetries in the forcing mechanisms during El Niño and La Niña events and how this affects frontal activity.

Although this study advances our understanding of low‐frequency variability in SST frontal activity, the use of a 37 years‐long time series of SST only allows for capturing a few El Niño events, and even fewer strong events. The relatively short time series also makes it difficult to identify the influence of other modes of variability, such as the Pacific Decadal Oscillation. As more data are collected and satellite time series are expanded, so will our ability to understand the relationships between SST frontal activity and climate variability.

## Supporting information

Supporting Information S1

## Data Availability

The data used in this study are publicly available at: GHRSST AVHRR‐only SST (NCEI, 2016; https://doi.org/10.5067/GHAAO-4BC02), MODIS SST (Ocean Biology Processing Group, 2015; https://doi.org/10.5067/MODSA-1D4D4), CCMP v2.0 surface vector winds (Mears et al., 2022; http://www.remss.com/measurements/ccmp/), sea level anomaly (Copernicus Climate Change Service, 2021; https://doi.org/10.48670/moi-00145), and the Oceanic Niño Index (NOAA Climate Prediction Center, 2015; https://psl.noaa.gov/data/correlation/oni.data).
