# Supplementary material for: Influence of the El Niño‐Southern Oscillation on SST Fronts Along the West Coasts of North and South America
Source: J Geophys Res Oceans. 2022 Oct 10;127(10):e2022JC018479. doi: 10.1029/2022JC018479 (PMC9787497; doi:10.1029/2022JC018479)
Supplement: Supplementary file 1 — Supporting Information S1 [file JGRC-127-e2022JC018479-s001.pdf]

Journal of Geophysical Research: Oceans

Supporting Information for

Influence of the El Niño-Southern Oscillation on SST fronts along the west coasts of North and South America

Caitlin M. Amos[1,2,*] and Renato M. Castelao[1]

[1]Department of Marine Sciences, University of Georgia, Athens, GA, USA

[2] Ocean Dynamics and Prediction Branch, Naval Research Laboratory, Stennis Space Center, MS, USA

*Corresponding author: Caitlin M. Amos (caitlin.amos.ctr@nrlssc.navy.mil)

Contents of this file:

Figures S1 to S3

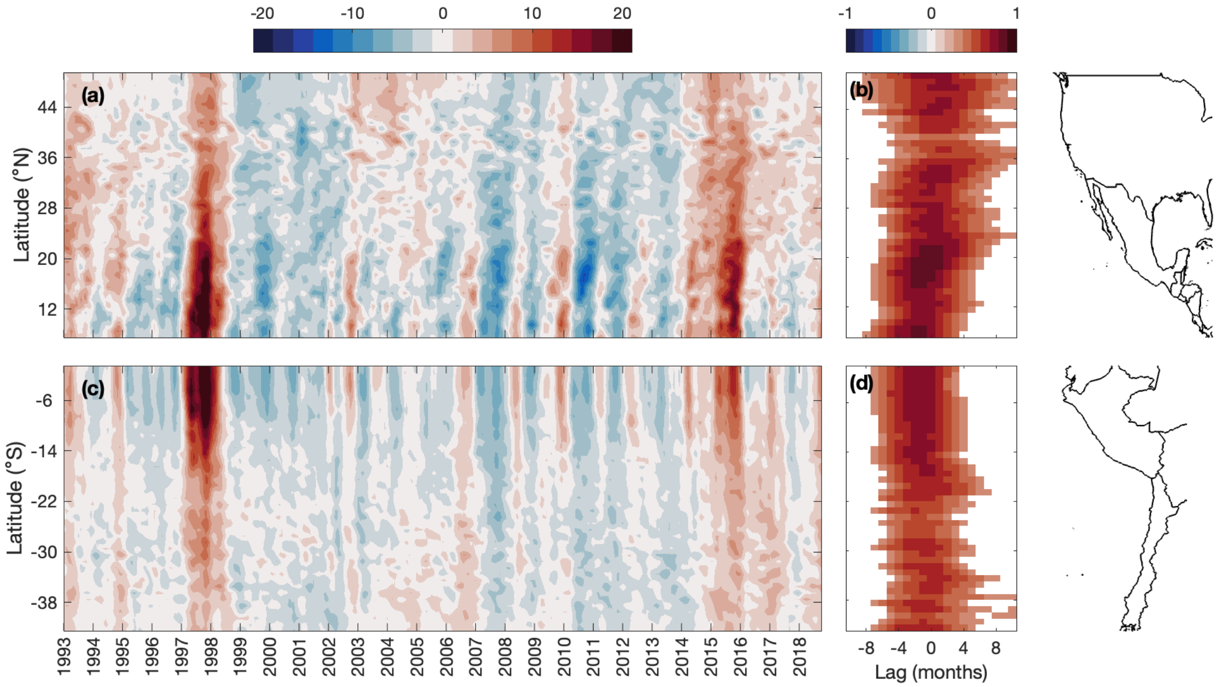

Figure S1. (a, c) The three-month running mean of anomalies of sea level anomaly (SLA; cm) computed by subtracting the climatological monthly mean (1993-2018) and the linear trend from each individual month using SLA produced and distributed by the Copernicus Climate Change Service. Anomalies were averaged within 0-300 km of the coastline in 1° latitude boxes. (b, d) Lagged correlation between SLA anomalies in panels a and c and the ONI (Figure 2e) at each latitude. The x-axis represents the months by which SLA lags the ONI (positive lag is defined as the changes in SLA occurring after the peak in ONI). Only significant correlations are shown. Coastlines for North/Central America and for South America are shown on the right.

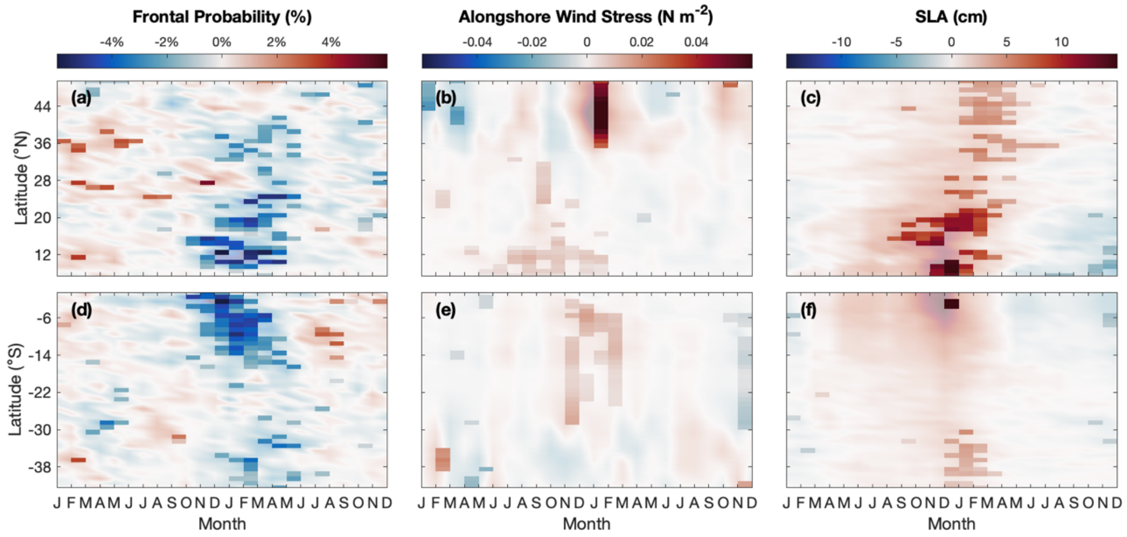

Figure S2. 2-year composite anomalies for moderate to strong El Niño events (defined as ONI ≥ 1°C for at least 3 consecutive months) in each 1° latitude box within 0-300 km offshore for (a, d) SST frontal probability (%), (b, e) alongshore wind stress (N m^{-2}), and (c, f) SLA (cm) along the west coasts of North/Central (a, b, d) and South (d, e, f) America. Light shading (transparency of 60%) indicates values that are not significant (p-value > 0.05). The composites for frontal probability, alongshore wind stress, and SLA included 8, 6, and 5 El Niño events, respectively. The same data are shown in Figure 4, but with significance levels determined using a p-value of 0.1.

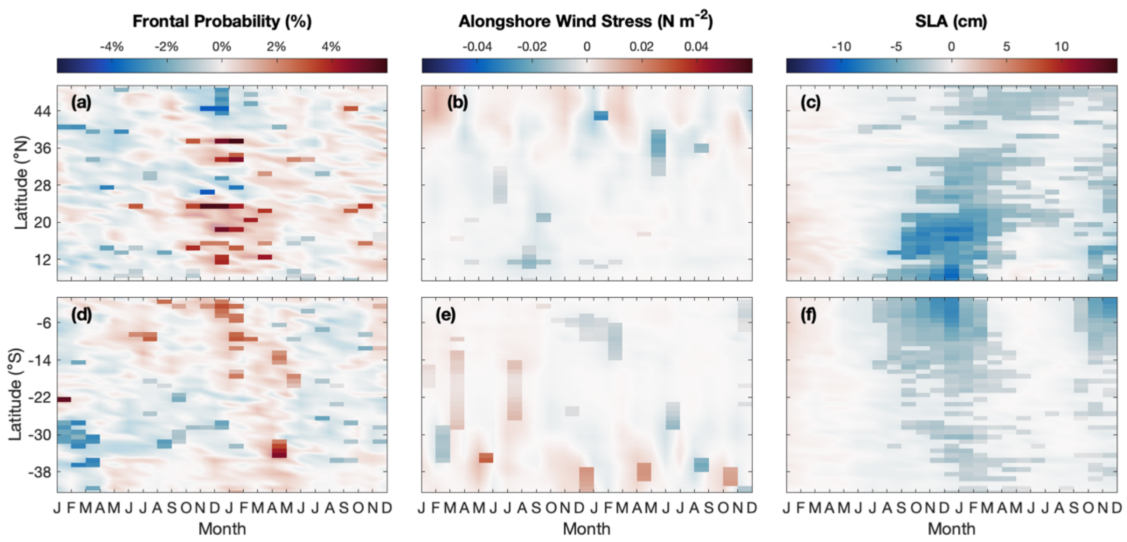

Figure S3. 2-year composite anomalies for moderate to strong La Niña events (defined as ONI ≤ -1°C for at least 3 consecutive months) in each 1° latitude box within 0-300 km offshore for (a, d) SST frontal probability (%), (b, e) alongshore wind stress (N m^{-2}), and (c, f) SLA (cm) along the west coasts of North/Central (a, b, d) and South (d, e, f) America. Light shading (transparency of 60%) indicates values that are not significant (p-value > 0.05). The composites for frontal probability, alongshore wind stress, and SLA included 7, 7, and 6 La Niña events, respectively. The same data are shown in Figure 5, but with significance levels determined using a p-value of 0.1.
